# Evidence of behavior-based utilization by the Asian citrus psyllid of a combination of UV and green or yellow wavelengths

**DOI:** 10.1371/journal.pone.0189228

**Published:** 2017-12-13

**Authors:** Thomson M. Paris, Sandra A. Allan, Bradley J. Udell, Philip A. Stansly

**Affiliations:** 1 Entomology and Nematology Department, Southwest Citrus Research and Extension Center, Immokalee, Florida, United States of America; 2 US Department of Agriculture, Agricultural Research Service, Center for Medical, Agricultural, and Veterinary Entomology, Gainesville, Florida, United States of America; 3 Department of Wildlife Ecology and Conservation, University of Florida, Gainesville, Florida, United States of America; United States Department of Agriculture, UNITED STATES

## Abstract

The Asian citrus psyllid, *Diaphorina citri*, vectors huanglongbing (HLB), the most serious disease affecting citrus globally. *D*. *citri* and HLB have spread to the major citrus growing regions of North America causing billions of dollars of damage in Florida alone. The visual behavior of *D*. *citri* is not well characterized and more knowledge is needed to improve attractive traps for monitoring and control of the *D*. *citri*. Bioassays were conducted to evaluate attraction to light transmitted through different colored filters. The addition of ultra-violet light (< 400 nm) enhanced attraction of *D*. *citri* to transparent visual targets made of green or yellow filters. However, attraction to blue targets was unaffected by UV light. This is the first study to demonstrate a phytophagous insect responding to a hue that is a combination of long and short wavelengths. Further testing is needed to determine how *D*. *citri* uses such discriminatory powers in the field. Our results further imply that *D*. *citri* utilize color vision, as the less intense yellow and green hues were chosen over white light. In summary, this research provides an increased understanding of *D*. *citri* visual behavior and can be used for the development of a more attractive *D*. *citri* trap than those currently available.

## Introduction

The most serious disease threatening the world-wide citrus industry is citrus greening or huanglongbing, caused by the pathogen *Candidatus* Liberibacter asiaticus [1,2]. This disease, vectored by the Asian citrus psyllid, *Diaphorina citri*, has resulted in a loss of US $4.54 billion in revenue and 6,600 jobs in Florida alone [3]. Since discovery of this species in Florida in 1998, populations of *D*. *citri* have not been controlled sufficiently to prevent the spread of huanglongbing throughout the citrus growing regions in Florida and to other citrus-growing regions of the United States [2,4]. There is no definitive cure for the disease in trees, so successful intervention relies on protection of new plantings of citrus from infection and prevention of expansion of the vector and disease to areas currently unaffected. Vector control, either through use of insecticides [5] or biological control agents [6], is essential for prevention of disease spread as well as mitigation of losses in intected trees [7,8]. Mitigation efforts are guided by surveillance which usually consists of colored sticky traps; however these traps are not optimized for detection at low population levels [9,10]. A better understanding of visually-guided behaviors in the context of color could contribute to enhancement of trap efficacy.

Phototaxis movement towards a visual target may be achromatically [11] or chromatically driven [12] and *Diaphorina citri* has been previously characterized with positive phototactic and negative geotactic behavior [13–17]. Attributes of a visual target include the chromatic characteristics of hue (peak wavelengths such as red, blue or green) and saturation (relative purity) as well as the achromatic characteristic intensity (relative intensity of energy) which together provide information relating to color perception for humans [18], however more insight on how these factors affect insect color vision is needed. Demonstration of color vision in insects requires the presence of at least two spectral receptor types to discriminate between colors, evidence of behavioral response independent of intensity [12,19] and the presence of a neural mechanism, such as color opposition for discrimination among spectral domains [19]. Optimally, conditioning studies can be used to demonstrate discrimination of a hue independent of other factors such as saturation or brightness [19,20,21].

Among phytophagous hemipterans, two or more photopigments have been confirmed in several species. Intracellular recordings of the compound eyes of the pea aphid, *Acyrthosiphon pisum*, documented three spectral receptor classes [22]. Extracellular recordings of the green peach aphid, *Myzus persicae*, showed maximum sensitivities at 320–330 nm, 440–480 nm and 530 nm [23]. Similarly, the cabbage aphid, *Brevicoryne brassicae*, possessed two peaks of spectral sensitivity at 350 and 520–530 nm respectively [24]. Extracellular recordings of *D*. *citri* eyes revealed peaks of sensitivity in the UV, blue and yellow/green region of the spectrum [25]. Dimensionality of color vision varies among insect species with honey bees having trichromatic vison [26,27] and the butterfly, *Papilio xuthus* having tetrachromatic vision [28]. The number of spectral receptors does not necessarily determine the dimensionality of color vision as discrimination of color based on behavior also needs to be considered. For example, *P*. *xuthus* has eight receptors yet appears to have tetrachromatic vision [29]. Phytophagous hemipterans generally are attracted to yellow and green targets with a strong preference for yellow visual targets documented for aphids [27,30], whiteflies [31–33] and psyllids [10,18,34]. In general, Hemiptera are not attracted to visual targets that reflect or emit light < 400 nm or > 600 nm [19,35]. Attraction to longer wavelength red light, however, has been demonstrated in the eucalyptus psyllids, *Anoeconeossa bundoorensis* and *Glycaspis brimblecombei* which feed on red anthocyanic leaves [36]. Attraction of phytophagous insects to yellow has been termed a "supernormal stimulus" in that attraction occurs to intensities of yellow exceeding those common in nature [37] and this attraction to yellow has been referred to as supernormal green [19]. The influence of degree of saturation of visual targets on behavior appears to differ by species by differing preferences between the mealy plum aphid *Hyalopterus pruni* [38] and the black bean aphid, *Aphis fabae* [39]. Attraction of *D*. *citri* has been primarily to yellow [10,16,34] and lime green [10,39] colored traps in the field and to yellow, green and UV-emitting LEDs (light emitting diodes) in the laboratory [40].

Many invertebrates have spectral receptors with peak sensitivity in the ultraviolet (UV) spectrum [12]. The ability to detect UV light has implications for color combinations not visible to humans, but in concept, may be similar to the color purple in human vision. Honey bees were the first insect species described as seeing a mixture of wavelengths from the long and short spectral domains [41,42] which was termed "purple" by Daumer [41]. Determining the parameters of honey bee color vision led to research into the diversity of flower colors present in the natural world. Of the 1,063 flowers surveyed that reflected light simultaneously stimulating short (UV) and long (green), or only short (UV) visual receptors of honey bees, only 15% attracted pollinators [43,44]. The nectar-laden flowers contrasted against a background of green leaves, brown soil, gray stones and several other inorganic materials, which appear gray to bees [44]. Wings reflecting both short (UV) and long (yellow) wavelengths are utilized by butterflies in interspecific and intraspecific sexual selection [45,46]. Unlike Hymenoptera and Lepidoptera, there is no evidence of attraction to a hue composed of a mixture of long and short wavelengths of the UV-VIS spectrum in Hemiptera. The objective of this study was to evaluate the role of UV light in the context of attraction to other wavelengths and in relation to the visual ecology of *D*. *citri*.

## Materials and methods

### Psyllids

Asian citrus psyllids, *D*. *citri*, were reared on orange jasmine, *Murraya paniculata* in a climate-controlled greenhouse at 29 ± 3°C under a photoperiod of 16:8 L:D (0600:-10:00 EST) using a combination of natural light and metal halide lamps. Plants were fertilized monthly with Miracle-Gro^®^ (The Scott’s Company, Marysville, OH)(N:P:K 24:8:16). Both rearing plants and randomly selected psyllids from the colony were evaluated using qualitative polymerase chain reaction (qPCR) to verify that neither plants nor insects were infected with *Ca*. L. asiaticus. Host plants were rotated into and out of colony cages at 4–6 week intervals to maintain plant health. Adult *D*. *citri* obtained from the colony for assays represented a mix of sexes, ages and physiological states presumably similar to field populations.

### Behavioral assays

**Fig 1:**
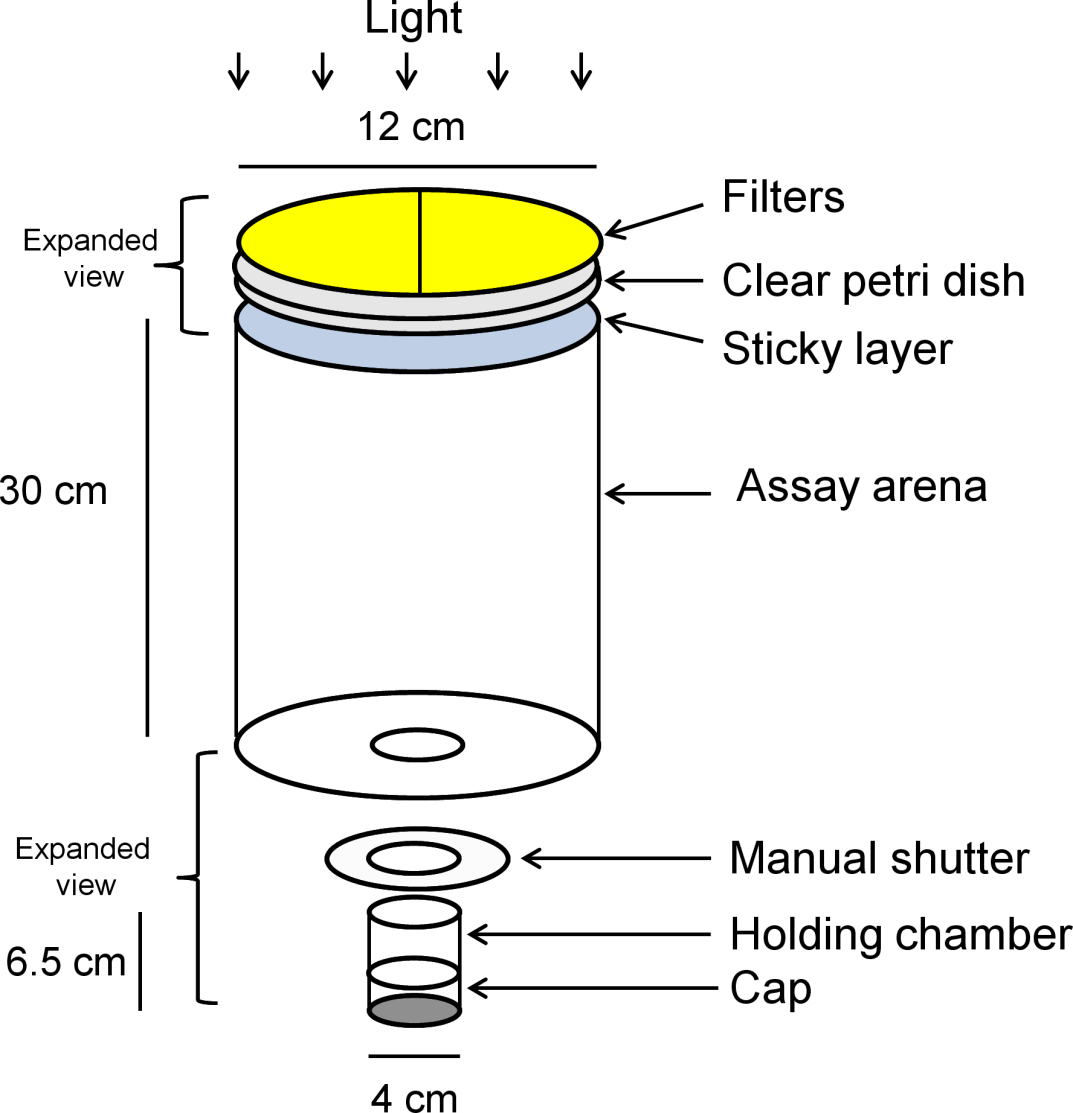
Schematic diagram of the bioassay apparatus for evaluation of phototactic psyllid responses to visual targets. A vial containing psyllids was placed within the holding chamber, the manual shutter closed for dark adaptation and the top covered with two semi-circular colored filters. The shutter was opened to allow psyllids into the arena and responding psyllids were captured on the sticky layer beneath the filters.

Visual preference responses to light transmitted through colored filters were evaluated using a two-choice visual target assay. The apparatus consisted of an arena with a holding chamber at the bottom and sticky translucent panels at the top for collection of responding psyllids (Fig 1). The arena consisted of a hollow black tube (12 cm wide x 30 cm tall) with a holding chamber (4 cm wide x 6.5 cm tall) attached to the center of the bottom of the area. Both the interior and exterior of the arena and chamber were painted flat black. A manual shutter (Polaroid MP-4, Eastman Kodak, Rochester, NY) was fastened to the bottom of the arena and the holding chamber was placed in the shutter opening. A black plastic cap (4.5 cm wide x 3 cm tall) fit over the bottom of the holding chamber. Assays were illuminated with a metal halide lamp (400 watts). The lamp was 55 cm above the top of the test arena and provided ca. 2000 lux with strong representation of both the UV and visible spectrum. The top of the test arena was covered with an inverted clear Petri dish (150 mm wide x 15 mm tall). The inside of the Petri dish bottom was coated with a thin sticky layer (Tangletrap^®^, Grand Rapid, MI). Visual targets consisted of two different dyed polyester filters mounted on the upper surface of the inverted Petri dish so that each filter covered one half (right or left) of the surface. The position of filters (right or left) was rotated between tests. The filters used for these tests were selected on the basis of their ability to transmit both UV and visible light. The filters included Roscolux^®^ yellow (#4530), green (#88), blue (#71), UV block (#3114) (94% transmission), and a neutral density filter (#3415) (0.15 optical density) (Rosco, Stamford, CT) and were obtained as sheets and cut to cover half of the Petri dish. To block UV transmission of light through colored filters, the UV-blocking filters were placed on top of colored filters. While the UV-blocking filters were essentially transparent to visible wavelengths, they reduced UV transmission to < 10% of the original intensity.

For assays, psyllids were aspirated into plastic vials (3 cm wide x 6 cm tall) with lids. Gaskets were placed around the outside of the vials so that the plastic vials fit snuggly in the holding chamber of the assay arena. Prior to an assay, the manual shutter was set in a closed position, the vial tapped to knock psyllids to the bottom, the cap removed and the vial quickly placed into the holding chamber. A black cap was placed on the bottom of the holding chamber so that psyllids were in complete darkness. For a test, psyllids were dark-adapted 15 min prior to the assay within the holding chamber. Preliminary tests determined that responses did not increase significantly after a 15 min dark-adaption period. For each assay, filters were placed on top of the test arena, psyllids were dark-adapted, the manual shutter was opened and psyllids were allowed 45 min to respond to the visual target. At the end of the assay, psyllids trapped on the sticky layer were considered responders and their sex determined. However, in the analysis, males and females were pooled as there was no significant difference between sexes in behavioral response (*P* > 0.05). Psyllids remaining in the chamber were considered non-responders; they were knocked down with CO_2_ so that their sex could be determined. Psyllids can be classified into different color morphs and both nonresponding and responding psyllids were categorized by abdominal color into the two predominate morphs (blue-green or orange-brown) [47]. All experiments were conducted between 07:00–18:00 hr [48]. Each test consisted of an average of 37.9 ± 0.60 psyllids and response was calculated as a proportion of the total responding. Assays were replicated 14 times.

### Spectral analysis

The spectral transmission through all of the filters tested was measured using a concave grating spectrometer (UV-VIS BLACK-Comet, StellaNet Inc, Tampa, FL). Transmission measurements were obtained using an integrating sphere to allow for observance of oblique angles of reflectance with measurements over the range of 350–700 nm. Reflectance spectra of biological materials in the psyllid environment (leaves, flowers, fruit, psyllid wings) were obtained to assess the presence of UV reflecting materials. Leaves of orange jasmine, *M*. *paniculata* (disease free) were obtained and segregated as flush (newly expanded leaves (stage 2) and mature leaves (hardened leaves (stage 7)) according to maturity stage categorization outlined in Michaud and Browning [48]. Flush and symptomatic leaves with yellow mottle from an orange plant (*Citrus sinensis* cv. Valencia) known to be huanglongbing diseased was obtained from the Citrus Research and Education Center, University of Florida for reflectance measurements. The HLB positive orange plant from which leaves were obtained had a cycle threshold value of 22.88 obtained using quantitative PCR methods described in Li et al. [49]. All measurements for transmission and reflectance were obtained using a xenon arc lamp (400 watt) as a light source with quartz light guides for light delivery and a magnesium oxide standard for reflectance. Three measurements were obtained and averaged for each measurement reported.

### Statistical analysis

Responses were calculated as the proportion of responding psyllids. Comparisons between responses in two-choice assays were evaluated for normality with Shapiro-Wilk test and if normal compared by paired t-test. If data were not normal, non-parametric log rank tests were applied (SigmaStat, San Jose, CA). The effects of covariates (sex, abdominal color) on overall response rate and comparisons of *D*. *citri* responding to the two choice assays were modeled using a generalized linear model (GLM) in R with a binomial distribution. The package dplyr was used in the analysis [50] within the R framework.

## Results

### Spectral analysis

**Fig 2:**
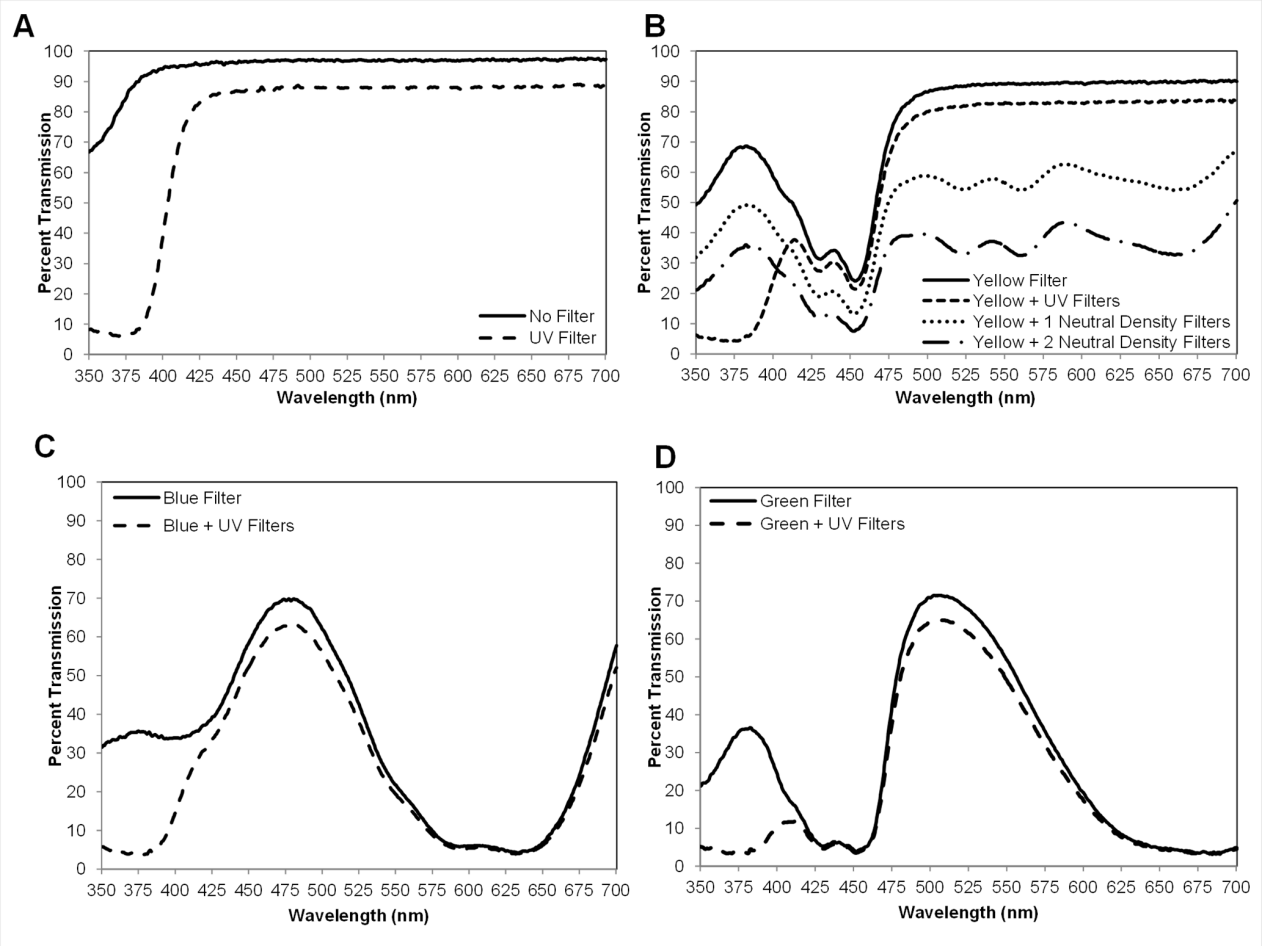
Transmission spectra through a clear Petri dish in conjunction with filters used in two-choice assays. (A) No filter vs UV-blocking filter. (B) Yellow filter alone, yellow with UV-blocking filter, and yellow filter with one and two neutral density filters. (C) Blue filter vs blue filter with UV-blocking filter. (D) Green filter vs green filter with UV-blocking filter.

**Fig 3:**
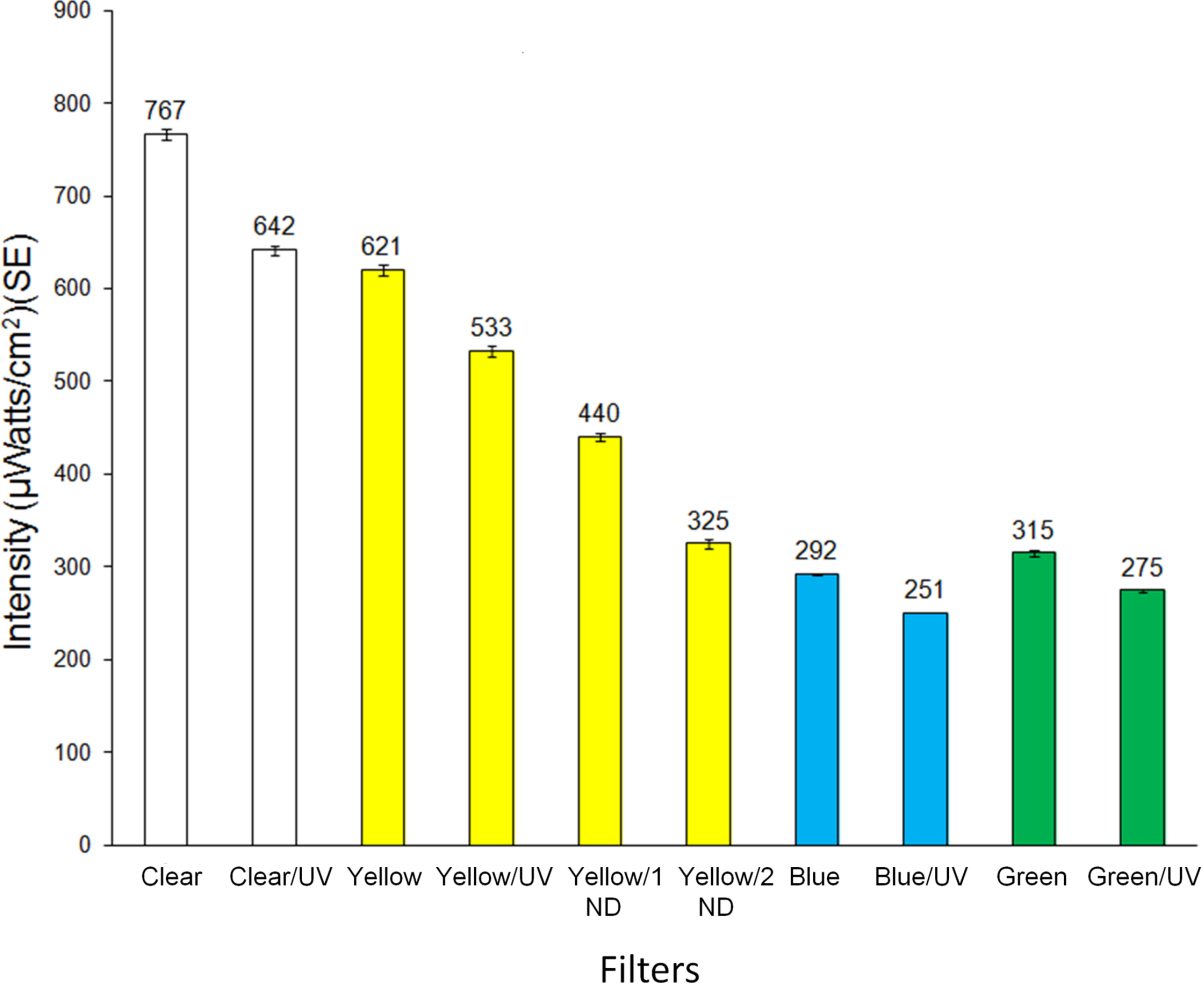
Intensity (μWatts/cm^2^) of light transmitted (350–700 nm) through single and double filters used in two-choice assays. UV = UV-blocking filters, ND = neutral density filters.

Transmission spectra for light that comprised the visual targets are presented in Fig 2. Transmission through the clear plastic (no filter) was high in the UV portion of the spectrum (>70%) and 93–100% in the visible (400–700 nm) portion of the spectrum (Fig 2A). The addition of the UV-blocking filter effectively removed UV light transmission through the clear filter with little UV transmission above 380 nm and about a 10% decrease in transmission for 400–700 nm (Fig 2A). The addition of the UV-blocking filter similarly reduced UV transmission for the yellow, blue and green filters (Fig 2B–2D) with < 10% intensity decrease for long wavelengths. Transmission of yellow light was further evenly reduced across the spectrum (350–700 nm) when either one or two neutral density filters were added in combination with the yellow filter (Fig 2B). Light intensities from 350–700 nm of all filters and filter combinations tested are presented in Fig 3. The reduction of overall intensity with the addition of UV-blocking filters resulted from reduced transmission in the UV region. Light intensity was highest through the clear and yellow filters with lowest transmission through the blue and green filters.

**Fig 4:**
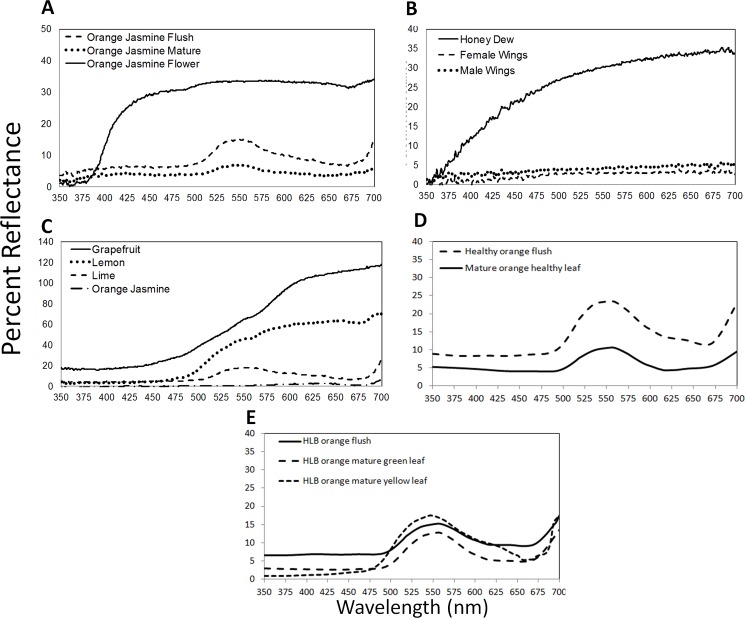
Reflectance spectra of vegetation, psyllids and fruit. (A) Young leaves/flush, mature leaves, and flower of orange jasmine *Murraya paniculata* (B) Wings of male and female Asian citrus psyllids and honeydew excreted by nymphs (C) Peel of grapefruit, lemon, lime and orange jasmine fruit (D) Healthy flush and mature leaves of orange, *Citrus sinsensis* (E) Huanglongbing-diseased flush, and ‘green’ and ‘yellow’ portions of the infected leaves of *C*. *sinsensis*.

Reflectance curves of biological materials present in the psyllid environment are presented in Fig 4. Immature leaves (flush) of orange jasmine were less saturated in color with higher overall light reflectance than mature leaves (Fig 4A). White flowers of *M*. *paniculata* reflected primarily above 400 nm with a small shoulder of UV reflectance from 380–400 nm (Fig 4A). Honeydew produced by *D*. *citri* nymphs reflected both UV and visible light with UV reflectance above 5% at ≥375 nm (Fig 4B). In comparison, the mostly transparent wings of male and female *D*. *citri* reflected light evenly from 374–700 nm (Fig 4B). The reflectance of the fruit of grapefruit, lemon, lime and orange jasmine was restricted primarily to the visible spectrum (Fig 4C). Flush of orange, *C*. *sinensis*, was higher in reflectance across all wavelengths (Fig 4D). Flush from diseased plants was similar to the non-diseased plants and the huanglongbing-infected yellow leaves were similar to flush but with slightly higher reflectance in the green and yellow.

### Behavioral assays

Overall 44.00 ± 0.01% of the *D*. *citri* released in the arena displayed positive phototactic responses to visual targets and were collected on the sticky targets. Response levels were similar between sexes (z = 0.17, df = 50, *P* = 0.52) except for the clear and UV-blocked clear visual targets which had a higher level of male non-responders (z = 2.80, df = 27, *P* = 0.005). Color of abdomen did not differ between responders and non-responders in treatments, except for fewer blue/green psyllids responding in the comparison of blue-UV blocked and yellow targets which had more blue/green (z = 2.51, df = 50, *P* = 0.01) and orange morphs (z = 2.58, df = 50, *P* = 0.01), respectively.

**Fig 5:**
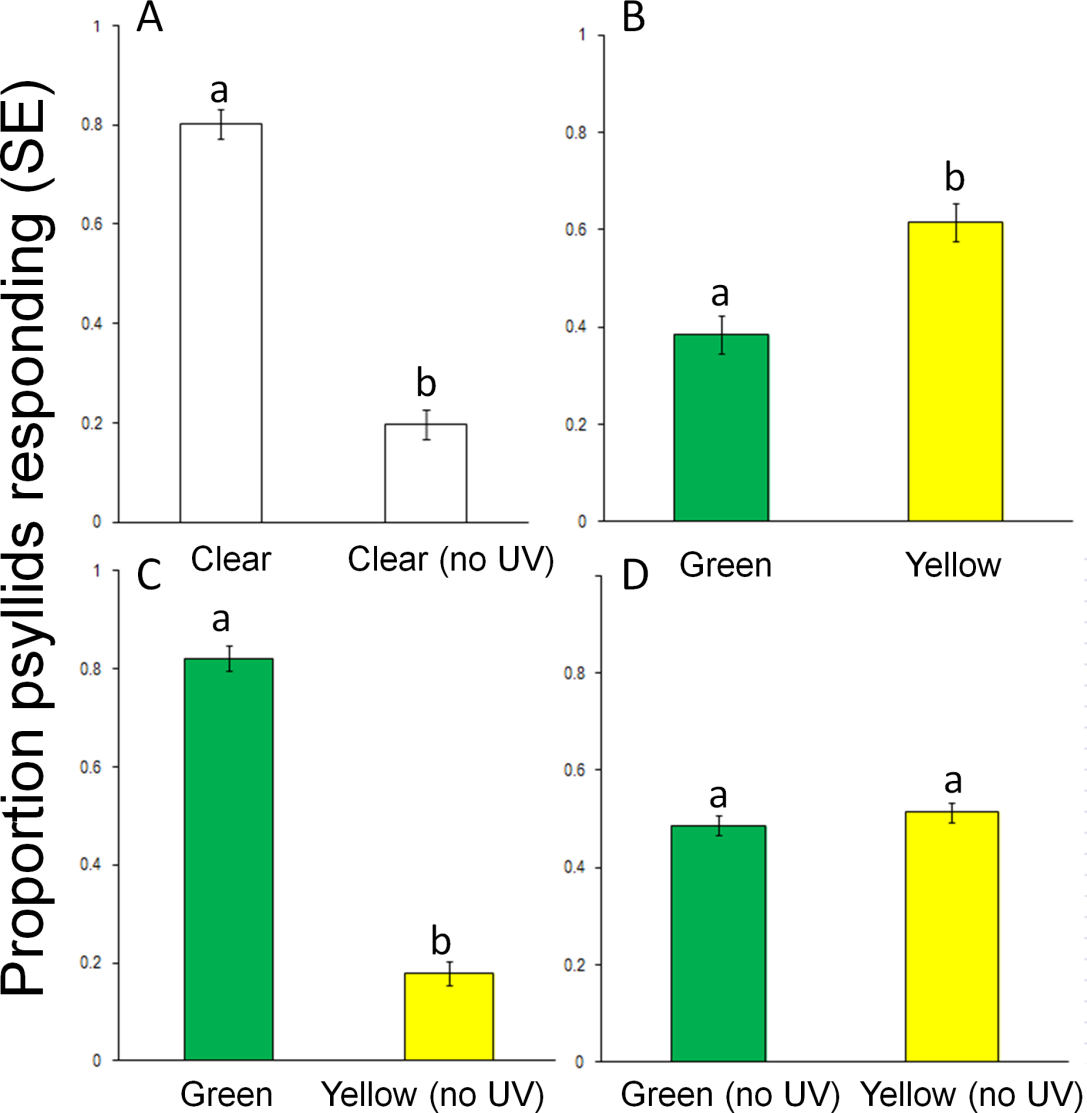
Responses (mean ± SE) of psyllids in two-choice assays to visual targets with clear, green, yellow and UV-blocking filters. (A) Clear filter vs. clear with UV-blocking filter, (B) Yellow filter vs. green filter, (C) Yellow and UV-blocking filter vs. green filter, (D) Green and UV-blocking filter vs. yellow and UV-blocking filter. Different letters above columns indicate significant differences (*P* < 0.05).

Preliminary studies comparing responses to two clear targets (white light) indicated no positional bias in the assay arena (data not shown) (t = -0.66, df = 26, *P* = 0.52). Responses to clear filters were high, but dropped approximately 4-fold with addition of the UV blocking filter, (t = 10.69, df = 26, *P* < 0.0001) (Fig 5A). Adding the UV-blocking filter reduced overall light intensity by 19%, with a 9% reduction between 400 and 700 nm (Fig 3). Significantly more *D*. *citri* were collected on yellow compared to green targets with no UV blocking (t = -4.00, df = 26, *P* < 0.0001) and twice the light intensity through the yellow filter (Fig 5B). However, significantly more psyllids responded to light from the green filter plus UV compared to yellow with UV-blocked, (t = 12.16, df = 26, *P* < 0.0001) (Fig 5C). Responses to yellow and green were equivalent to when UV was blocked from both targets despite twice the light intensity through yellow filters (t = -0.89, df = 26, *P* = 0.38) (Fig 5D).

**Fig 6:**
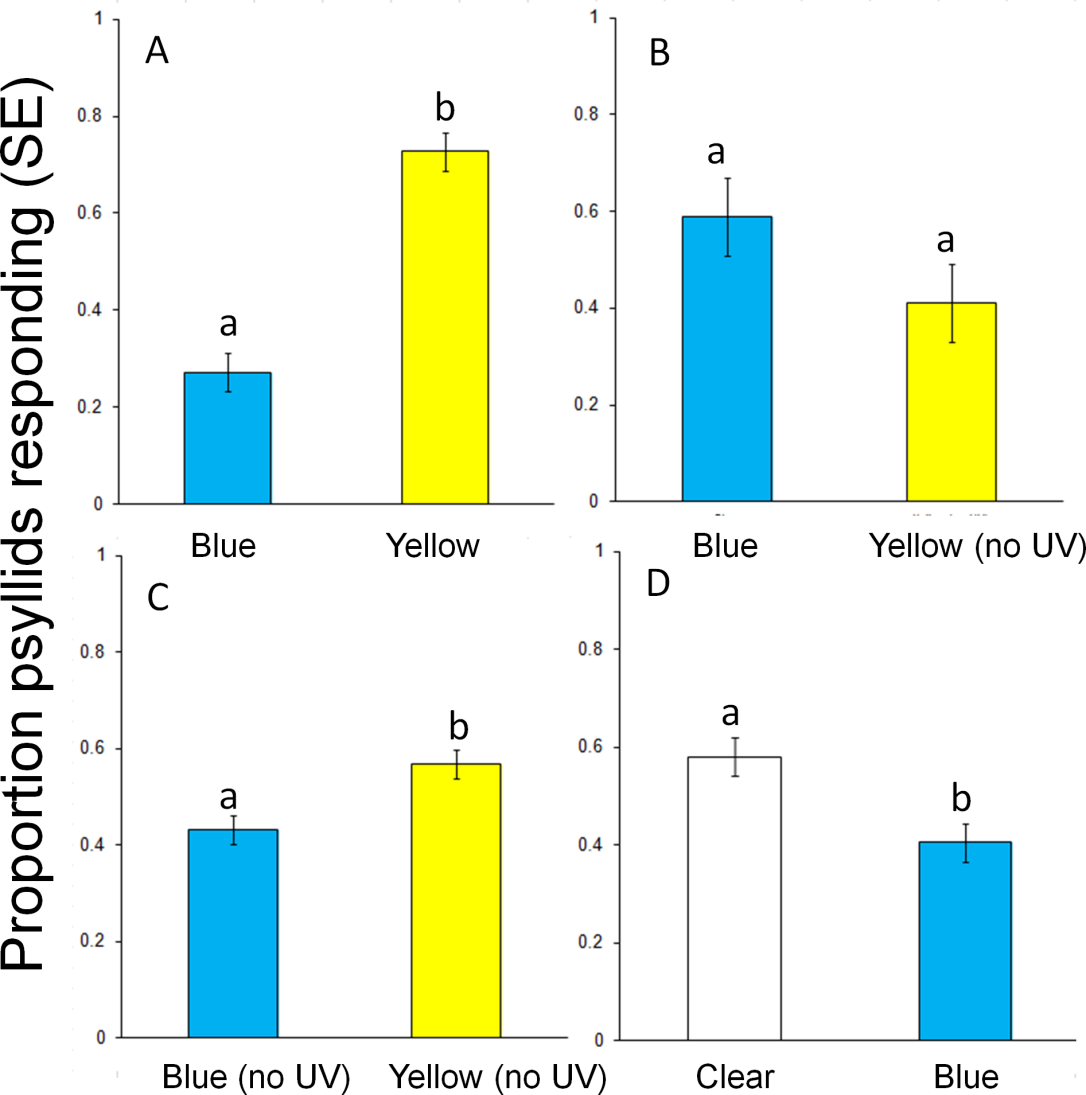
Responses (mean ± SE) of *D*. *citri* in two-choice assays to visual targets with blue, yellow and UV-blocking filters. (A) Yellow filter vs. blue filter, (B) Yellow and UV filter vs. blue filter, (C) Yellow and UV filter vs. blue and UV filter, (D) Clear filter vs. clear with blue filter. Different letters above columns indicate significant differences (*P* < 0.05).

In a second series of tests, the role of UV light in attraction to yellow and blue targets was examined. Responses to yellow targets were significantly greater (~ 3-fold) than to blue targets (t = -6.73, df = 26, *P* < 0.0001) (Fig 6A) with no UV blocking and yellow targets having twice the intensity of blue targets. However, blocking UV light from the yellow filter decreased response which was then similar to the unblocked blue target (t = 0.95, df = 26, *P* = 0. 35) (Fig 6B) even though the yellow was still 1.75x more intense. Responses were again greater for yellow when UV light was blocked from both targets, (t = -3.21, df = 26, *P* < 0.01) (Fig 6C) with 1.9X greater light intensity for the yellow target. Responses to the yellow target without UV (Fig 5B and 5C) were 1.5 less than the yellow target with UV (Fig 6A). Thus, yellow without a UV component was more attractive to psyllids than blue without the UV component, possibly as a function of wavelength or light intensity. Phototactic responses of psyllids were stronger to light white light from the clear filter (t = 2.53, df = 26, *P* = 0.02) (Fig 6D) blue light which was 2.5X less intense.

**Fig 7:**
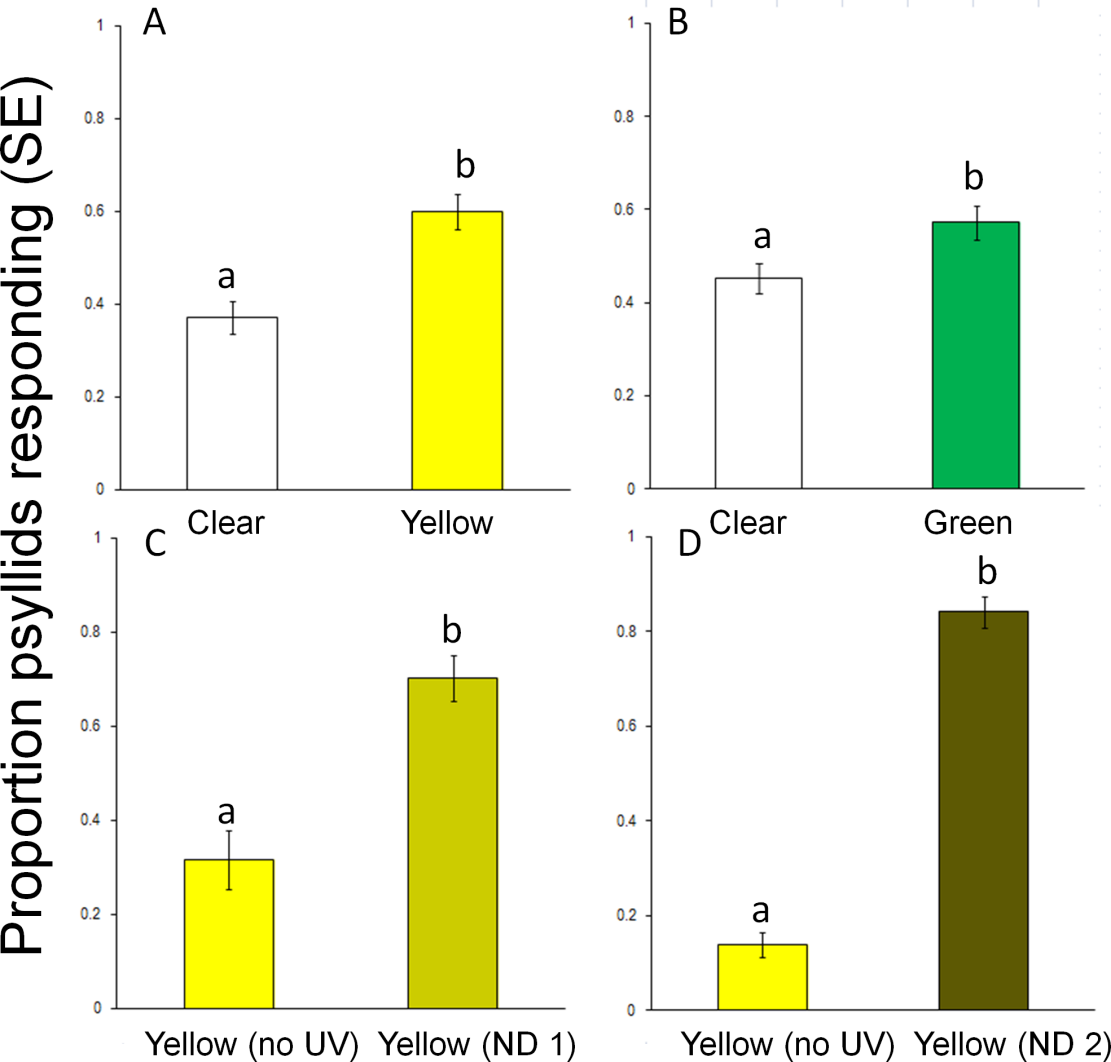
Responses (mean ± SE) of *D*. *citri* in two-choice assays to visual targets with clear, yellow, green and UV-blocking filters. (A) Clear filter vs. yellow filter, (B) Clear filter vs. green filter, (C) Yellow and UV-blocking filter vs. yellow with neutral density (filter) and UV filter, (D) Yellow and UV-blocking filter vs yellow with two neutral density filters. Different letters above columns indicate significant differences (*P* < 0.05).

In a third series of tests, the role of UV light and light intensity in attraction to yellow filters was examined. In a comparison between a yellow target and clear target (both with UV), phototactic responses were stronger to light from the yellow target compared to the clear target (t = -4.35, df = 28, *P* < 0.001) (Fig 7A) despite the clear target being 24% brighter. This provides additional evidence of a wavelength-based preference. Similarly, the unblocked green target was significantly more attractive than the unblocked clear target which was 1.4-fold brighter (t = -2.46, df = 26, *P* = 0.02) (Fig 7B). Light intensity from the clear filter (766.6 ± 6.1 uwatts/cm^2^/s) was more intense than through the yellow (620.5 ± 6.1 uwatts/cm^2^/s) or green (315.1 ± 3.2 uwatts/cm^2^/s) filters, however responses were greater to specific wavelengths and not associated with light intensity. To eliminate the effect of light intensity on target choice a comparison was made between a yellow filter with UV light blocked and a yellow filter with light intensity reduced by one or two neutral density filters which decreased light intensity by 41% and 91% respectively. Nevertheless, yellow targets that did not contain UV light were less attractive than yellow targets with lower intensity through use of one (t = 10.89, df = 26, *P* < 0.0001) or two (t = 24.39, df = 26, *P* < 0.001) neutral density filters (Fig 7C and 7D). The preference by *D*. *citri* for yellow targets consisting of long and short wavelengths (yellow and UV light) even when at a lower light intensity over yellow targets at higher light intensities but without UV components provides clear evidence of the behavior role of the combination of long and short wavelengths).

## Discussion

The strong attraction of *D*. *citri* adults to targets consisting of a combination of ultraviolet with yellow or green light indicates that UV can play an important role in attraction of *D*. *citri* attraction. Removal of the UV component of light from the visual targets reduced *D*. *citri* attraction for each color tested demonstrating that there was discrimination of UV separate from white or long wavelength light. This is the first report of the use of two combined wavelengths that are discriminated separately affecting phototactic behavior in *D*. *citri*. The presence of at least three different photoreceptors in *D*. *citri* representing the UV, blue and green/yellow portions of the spectrum [25] supports the capability of this species to discriminate between UV and yellow light.

Responses of *D*. *citri* to UV light appear to involve both positive and negative phototaxis. Adult *D*. *citri* exhibited strong positive phototactic responses to UV-emitting LEDs with maximum response to LEDs at 375 nm [40]. Use of metalized UV-reflective mulch resulted in reduction in infestation of young citrus trees by *D*. *citri* as well as reduction of infection with CLas [51]. Similar to studies with other insects, the reduction of infestation was assumed to be due to the disruption of normal host plant location behavior of day-flying insects by reflection of UV light from the substrate. Among other hemipterans, photoaxis is also affected by UV. Aphids are strongly attracted to UV light from the sky when initiating flight but repelled by UV when alighting [52]. The white fly, *Trialeurodes vaporariorum*, exhibits positive phototaxis towards UV targets (< 400 nm) and a strong settling response on green visual targets (550 nm) [31,32]. In terms of flight behavior, the autumn form of the aphid, *A*. *fabae*, was more prone to longer flights towards a white (UV-VIS) light than to settle on a green target [30].

In this study, evidence of innate preference for both yellow and green was provided by the selection of lower intensity colored targets over clear higher intensity targets. However, evidence of discrimination between yellow and green was not obtained nor was it the objective of this study as targets used were broad bandpass filters that allowed transmission of both short and long wavelengths with overlap in wavelengths transmitted. Preference by *D*. *citri* for yellow and green wavelengths has been reported previously [10,34,39,40] and is typical of phytophagous Hemiptera [35,53]. In a study on attraction to different colored LEDs, *D*. *citri* were equally attracted to light emitted from UV, green and yellow LEDs [40].

Unsaturated (high intensity) yellow reflected from visual targets has been demonstrated to be more effective in attraction of aphids than lower intensity saturated yellow targets [38]. In our study, yellow visual targets incorporating a combination of long and short wavelengths were more attractive than yellow alone. Even with the addition of neutral density filters and a corresponding decrease in light intensity, yellow light containing UV but at an overall lower intensity was still more attractive to *D*. *citri* than yellow light with the UV blocked. The strong preference for yellow light in *D*. *citri*, and quite possibly throughout Hemiptera, may be related to the interaction between long and short (UV) wavelengths. Host plants are known to be rendered less visible or attractive to aphids, whiteflies and thrips when short wavelengths of light (UV) are blocked in greenhouses [54,55] or field crops [56,57]. Similarly, when natural UV light components are altered such as being reflected from reflective mulch on the ground, attraction to host plants is decreased as demonstrated for psyllids, aphids, thrips, and whiteflies [56–60]. It is possible that the lack of UV alters propensity for flight and this requires further research.

Color vision in insects is characterized as the ability to discriminate between two spectrally-defined targets independent of intensity and is based on the presence of at least two photoreceptors, behavioral evidence of discrimination and a mechanism for detection [18]. Extracellular spectral sensitivity data indicate the presence of at least UV, blue and green photoreceptors in *D*. *citri* [25]. Data from our study provide evidence that *D*. *citri* utilize both green/yellow and UV-sensitive photopigments for discrimination of targets in terms of phototactic behavior. Perception of blue visual targets is supported by decreased attraction observed to blue LEDs [40] and conditioning experiments [61,62]. While the essential components supporting color vision in *D*. *citri* are present, the processing mechanism is not entirely clear. Color opponency (color antagonism) is a proposed mechanism for color vision that has been applied to vision in the aphid species *M*. *persicae* and *A*. *fabae* [19,62–64] that entails discrimination of two or more wavelengths, with at least one excitatory and the other not and possibly antagonistic. For *M*. *persicae*, UV light was negative based on data from Moericke [27] indicating that addition of a UV-blocking filter reduced response to yellow. Blue light was considered negative and yellow considered attractive. The opponent mechanism was described by an equation summing the interactions [18]. Based on behavioral responses from *D*. *citri*, which differ from *M*. *persicae*, a different color opponency mechanism is proposed for *D*. *citri*;

$$E_{\mathrm{opp}}=+{\mathrm{aE}}_{\mathrm{UV}}\text{-}b_{\mathrm{EB}}+{\mathrm{cE}}_{\mathrm{GY}}$$

where E_UV_ and E_GY_ represent excitation resulting from the UV and yellow/green putative receptor regions, respectively, and E_B_ represents the inhibition or repellency from the blue receptor regions. The letters a—c represent the unknown weighting factors for each receptor and E_opp_ represents the total sensory excitation for *D*. *citri* as a result of a visual stimulus. Further research is required to evaluate this mechanism.

Overall there were few differences between sex and color morphs of *D*. *citri* in phototactic behavioral to colored targets. The higher number of male nonresponders to clear and UV-blocked clear targets may be related to lack of innate phototactic response to non-colored targets. Adult *D*. *citri* are present in brown/gray/orange and blue/green color morphs with the latter associated with the highest fecundity, larger mass [46,65–67], greater resistance to insecticides [68], long-duration flight [69] and are considered an indicator of *D*. *citri* infestation of new flush present in the spring [70]. With increasing evidence of the association of blue/green morphs with establishment of infestations in the field, their lower responses stimuli that do not elicit positive phototaxis (blue and UV-blocked targets) is not surprising.

The strong attraction of *D*. *citri* to yellow may facilitate visual preference for attraction to diseased citrus leaves as the spectral characteristics of yellow are particularly prevalent from immature and senescing plant leaves as well as diseased plants [30,71,72]. The asymmetrical yellow mottling on citrus shoots characteristic of huanglongbing is caused by the phloem-limited bacteria *C*. L. asiaticus [73]. In terms of a visual target, ‘*Ca*. L. asiaticus’-positive plants are more yellow and less saturated in color than ‘*Ca*. L. asiaticus’ negative plants [74–76], thus appearing more like flush. Furthermore, *Citrus sinensis* leaves positive for *Ca*. L. asiaticus possess an altered plane of polarization for yellow (591 nm) wavelengths, reflecting increased accumulation of starch [77,78] The increased yellow, decreased color saturation and altered polarization may contribute to the visual differentiation of huanlongbing- positive from negative plants by *D*. *citri* in the field [77,78]. The role of polarization in *D*. *citri* response remains unknown.

The reduction of phototactic behavior in this study by the removal of UV light from yellow and green targets has direct relevance to management strategies for psyllids in enclosed factilities. UV-blocking film has previously been shown to reduce infestation of aphids, whiteflies and thrips in greenhouses and other facilities through reduction of host plant finding behavior [57,79]. Similarly, use of UV-blocking film was recently associated with reduction of flight initation and host plant finding abilities of *D*. *citri* in screen houses [55].

It is unclear how sensitivity to the stimulus of combined wavelengths of UV and yellow light is involved in the visual ecology of *D*. *citri*. In general, leaves absorb UV light with the majority of leaves reflecting and transmiting <10% of UV light [80,81]. Citrus leaves are glabrous and varieties such as *Citrus paradisi* reflect 7–8% of UV light (330 nm) [81]. Of the materials examined in this study including psyllid wings, and the leaves, flower and fruit of orange jasmine and citrus fruit, very little UV light was reflected. In contast, excreted honeydew clearly reflected UV light at a low level and this would contrast against darker foliage, although this is not known to be associated with psyllid attraction. While a role for UV in orientation based on yellow-green reflection from vegetation remains unclear, detection of UV-absorbing vegetation against an UV-emitting sky would enhance contrast and presumably detection of potential host plant material. Adult *D*. *citri* appear to have an innate preference for vegetation at the edge of groves [82] where high levels of illumination including UV are present [83]. Adult *D*. *citri* prefer younger flush shoots [84] which have higher reflectance values, particularly in the green and yellow portions of the spectrum [85]. Additionally, leaves with a thin epidermis such as new flush tissue are known to reflect more UV than leaves with thick epidermis (older leaves) [85]. Possibly the slightly higher levels of UV reflected from yellow-green new flush aid in preferential detection by *D*. *citri* of psyllid infestation on plants in these locations. While it is clear that UV and yellow light can play a role in phototactic behavior of *D*. *citri*, further research is required to clarify the role in the context of psyllid behavior.

## Supporting information

S1 Dataset Original data. The data file contains data for experiments described in the text.
(XLSX)
